# Diversification of land plants: insights from a family-level phylogenetic analysis

**DOI:** 10.1186/1471-2148-11-341

**Published:** 2011-11-21

**Authors:** Omar Fiz-Palacios, Harald Schneider, Jochen Heinrichs, Vincent Savolainen

**Affiliations:** 1 Division of Ecology and Evolution, Imperial College London, Silwood Park Campus, Ascot SL5 7PY, UK; 2 Department of Systematic Biology, Evolutionary Biology Centre, Uppsala University, Norbyvägen 18 D, Uppsala 75236, Sweden; 3 Department of Botany, Natural History Museum, London SW7 5BD, UK; 4 Albrecht-von-Haller-Institute of Plant Sciences, Georg-August University, 37073, Göttingen, Germany; 5 Royal Botanic Gardens, Kew, Richmond TW9 3DS, UK

## Abstract

**Background:**

Some of the evolutionary history of land plants has been documented based on the fossil record and a few broad-scale phylogenetic analyses, especially focusing on angiosperms and ferns. Here, we reconstructed phylogenetic relationships among all 706 families of land plants using molecular data. We dated the phylogeny using multiple fossils and a molecular clock technique. Applying various tests of diversification that take into account topology, branch length, numbers of extant species as well as extinction, we evaluated diversification rates through time. We also compared these diversification profiles against the distribution of the climate modes of the Phanerozoic.

**Results:**

We found evidence for the radiations of ferns and mosses in the shadow of angiosperms coinciding with the rather warm Cretaceous global climate. In contrast, gymnosperms and liverworts show a signature of declining diversification rates during geological time periods of cool global climate.

**Conclusions:**

This broad-scale phylogenetic analysis helps to reveal the successive waves of diversification that made up the diversity of land plants we see today. Both warm temperatures and wet climate may have been necessary for the rise of the diversity under a successive lineage replacement scenario.

## Background

It is believed that climate change is one of the main factors affecting global biodiversity [1-3]. During the history of life, fluctuations of the world's climate have most likely caused major extinctions [4] and led to the development of new ecosystems, promoting new biotic interactions and the evolution of novel adaptive traits. The dynamics of such diversification events can be studied based on phylogenetic trees dated with fossils. Here we focus on land plants. The origin and diversification of land plants has intrigued biologists for centuries. According to the fossil record, land plants diverged from green algae before 475 million years ago (Ma; first land plant fossil) and led to the major clades found today [5,6]. These are liverworts (74 families, ca. 6,000 spp. [7]), mosses (112 families, ca. 12,000 spp. [8,9]), hornworts (five families, ca. 150 spp. [10]) and tracheophytes. The latter include ferns (45 families, ca. 9,000 spp. [11]), lycophytes (three families, ca. 1,200 spp. [12]), and seed plants, which in turn are separated into gymnosperms (14 families, ca. 1,000 spp. [13]) and angiosperms (456 families, ca. 260,000 spp. [13]).

There are various possible scenarios to describe the processes that influenced land plant diversification throughout geological time. One frequently proposed scenario is based on a successive replacement of ancestral lineages by more derived lineages, which in turn evolved similar habits (e.g., tree-like structure for forested ecosystems), and diversified to fill up the niches left empty after the extinction of the 'previous' taxon. In this kind of scenario, extant taxa of liverworts, mosses, and ferns, are considered to be relicts of previous radiations [14]. An alternative scenario suggests a coincidence between diversification events in each of the extant land plant lineages instead of a 'continuous replacement' idea. In this case, the majority of extant diversity is either the result of recent radiation events or of a long accumulation of species diversity throughout a taxon's history [14]. External factors, such as the break-up of continents and climate fluctuations, are prominent factors influencing the branching of the tree of life.

In this study we ask two questions: (1) Do we find evidence for non-constant rate of diversification in land plants? (2) Are major shifts of diversification rates, if any, correlated with some major external factors such as global climate warming or cooling?

## Results

We inferred the divergence times of over 98% of all families of land plants in a single phylogenetic analysis based on multiple genes from two genomes (Additional file 1; TreeBase study ID S11106). The topology and divergence times retrieved from the various analyses are broadly congruent with previous studies with limited sampling [15-17]. All major lineages of land plants as well as the relationships among them were supported (bootstrap support > 74%) being mosses (62%), lycophytes as sister to seed plants (68%) and hornworts as sister to mosses (47%) the clades with lowest bootstrap values (TreeBase study ID S11106). This topology is congruent with the analysis using the three most complete markers (18S, rbcL and atpB; 6.3% missing data).

The tree was calibrated using multiple fossils. In one of the calibration procedures, we also constrained the age of angiosperms to a maximum of 130 Ma following Brenner [18] (hereafter the constrained tree). The estimated crown age of land plants was 544.7 Ma (confidence interval [C.I.] = 563.1-536.5) and that of angiosperms was 267.6 Ma (C.I. = 289.9-263.2; Additional file 2; TreeBase study ID 11106) whereas for the constrained tree we obtained a crown age for land plants of 510.8 Ma (C.I. = 512.9-475.5).

**Figure 1:**
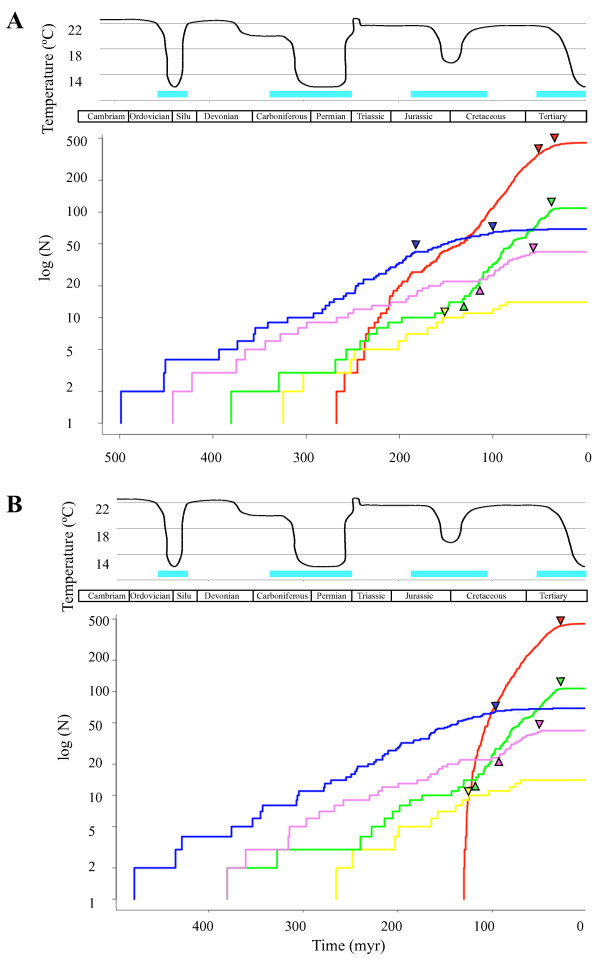
**Lineage Through Time plot**. Lineage Through Time (LTT) plot for liverworts (blue), mosses (green), ferns (purple), gymnosperms (yellow) and angiosperms (red) with indication of average global temperature [22] and cool climate modes (blue bars [21]). Triangles pointing up or down indicate diversification rate shifts as detected with LASER (increasing or decreasing, respectively, see Methods). (A) unconstrained tree; (B) constrained tree (i.e., angiosperms not older than 130 Ma). The y-axis indicates the number of lineages N on a logarithmic scale.

We produced lineage through time (LTT) plots for both time estimations, presented in Figure 1. These show a roughly constant rate of lineage increase (at least for the family-level studied here), although for angiosperms, ferns and mosses some acceleration is apparent since the Cretaceous, while for liverworts and gymnosperms a slowdown is observed (Figure 1).

**Figure 2:**
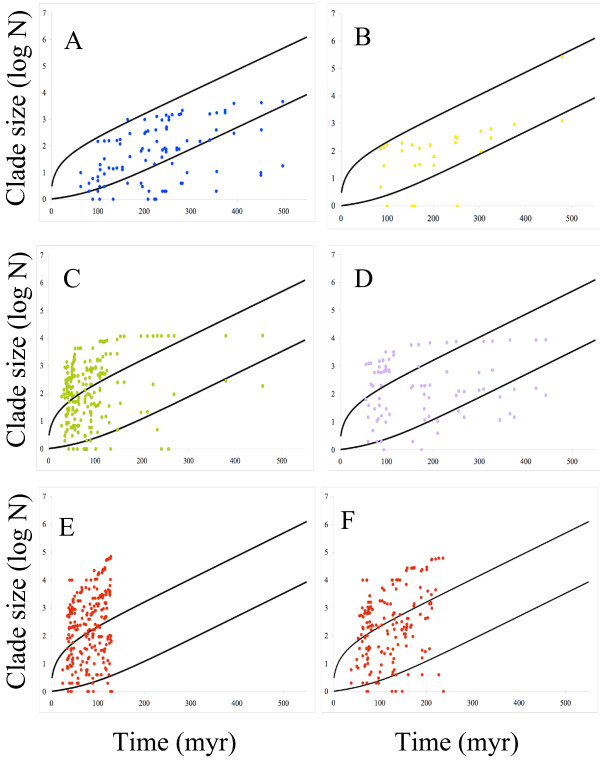
**Clade-size through time plot**. Clade-size through time plot with indication of the 95% C.I. depicted from the rate of diversification of land plants as a whole and assuming some background extinction (see Methods): (A) liverworts, (B) gymnosperms, (C) mosses, (D) ferns (E) angiosperms unconstrained tree (F) angiosperms constrained tree.

A congruent pattern is obtained when we explore the data applying a high level of background extinction using a methodology developed by Magallón & Sanderson [19]. Figure 2 shows sizes of the major clades against a 95% confidence interval of background diversification through time for land plants as a whole. In recent times, most clade sizes for mosses (Figure 2C), ferns (Figure 2D) and angiosperms (Figures 2E &2F, the former being the tree from a constrained analysis) fall above these confidence intervals.

Using a topology-based test of diversification [20], a total of 135 significant rate shifts were identified, with a similar figure found for the constrained tree (139; Table 1). The inclusion of 11 families with no DNA data resulted in the identification of just one more shift in diversification, i.e. on the branch leading to Balanophoraceae. We then explored the concordance of these shifts with the major cool and warm climatic modes [21] and we found some striking correlations. The majority of shifts in diversification rates in angiosperms, ferns, and mosses coincide with the last warm climate mode (Table 1). For liverworts, the highest number of shifts (5) took place in another warm climate mode (184-252 Ma; Table 1). For gymnosperms, only one shift in net diversification occurred, but in this case, during a cool period. This pattern appears to also hold if we compare the timing of shifts in diversification rates with the more continuous global temperature change presented in Scotese [22] (Additional file 3).

**Table 1:** Number of significant shifts in net diversification rate (topology-based method of Moore et al.[20]) with indication of cool (black) and warm (bold) climate modes of the Phanerozoic [21].

Climate modes	0-54		55-105		106-183		184-252		253-333		334-420		421-458		Total	
Angiosperms	23	*29*	**54**	***52 ***	16	*20 *	**6**	**-**	-	-	**-**	**-**	-	-	99	*101 *
Ferns	-	-	**5**	***4 ***	1	*1 *	**1**	***1 ***	1	*1 *	**-**	***2 ***	2	-	10	*9 *
Mosses	2	*7 *	**6**	***8 ***	3	*2 *	**-**	**-**	-	-	**-**	**-**	-	-	11	*17 *
Gymnosperms	-	-	**-**	**-**	1	*1 *	**-**	**-**	-	-	**-**	**-**	-	-	1	*1 *
Liverworts	-	-	**-**	**-**	2	*1 *	**5**	***4 ***	4	*4 *	**2**	***1 ***	1	*1 *	14	*11 *
Total	25	*36 *	**65**	***64 ***	23	*25 *	**12**	***5 ***	5	*5 *	**2**	***3 ***	3	*1 *	135	*139 *

Bold number columns correspond to periods of rate increases. Time unit is Mya (million years ago). For each lineage the bottom row (*italics*) corresponds to the constrained tree.

Using another diversification test that take into account branch lengths (i.e., LASER [23]), constant rates of lineage diversification were rejected for all major subclades. In gymnoperms, the best model was one with a rate shift occurring ca. 154 Ma, corresponding to a decrease in diversification during a cool climate mode (Table 2, Figure 1A). In the other subclades, two-variable rates were favoured (Table 2, Figure 1A). In angiosperms, two consecutive slowdowns in diversification were identified for the current cool climate mode. In liverworts, a similar pattern was encountered but decreases in rates of diversification occurred firstly in a cool climate mode (ca. 184 Ma) and secondly during a warm climate mode (ca. 99 Ma). In ferns and mosses, we first observe two increases in diversification during a cool climate mode (ca. 106 and 133 Ma, respectively, Table 2, Figure 1A). Subsequently, two decreases took place 60 Ma (warm mode) for ferns and 35 Ma (cool mode) for mosses (Table 2, Figure 1A). With the constrained tree, the pattern is similar for gymnosperms (Figure 1B, Additional file 4). In the case of angiosperms and liverworts, only one decrease was retrieved about 34 (cool mode) and 99 Ma (warm mode) ago, respectively (Figure 1B, Additional file 4). This pattern is similar to that obtained in the unconstrained tree for mosses and ferns (Figure 1B, Additional file 4), although this time fern diversification increases during a warm mode (ca. 93 Ma) and decreases during a cool mode (ca. 52 Ma; Figure 1B, Additional file 4).

**Table 2:** LASER analysis using a constant-rate birth-death model with no extinction (a = 0) against variable-rates models with 2 and 3 rates (r) and 1 or 2 time shifts given for best fitting model (ts; time unit is million years ago).

	Birth-death model (a = 0)	2-rates model (r1, r2, ts)	3-rates model (r1, r2, r3, ts1, ts2)
Angiosperms			
AIC	274.8786	-6.7234	-43
Delta AIC	0	281.602	317.40126
Ts1			27.53
Ts2			52.43
Ferns			
AIC	233.6887	232.7399	231.2457
Delta AIC	0	0.9488	2.443
Ts1			60.02
Ts2			106.39
Mosses			
AIC	388.785	306.4588	301.9242
Delta AIC	0	82.3262	86.8608
Ts1			35.19
Ts2			132.65
Gymnosperms			
AIC	113.2583	107.9801	109.1307
Delta AIC	0	5.2782	4.1276
Ts		153.76	
Liverworts			
AIC	416.5246	365.2789	360.1666
Delta AIC	0	51.2457	56.358
Ts1			99.52
Ts2			183.89

**Figure 3:**
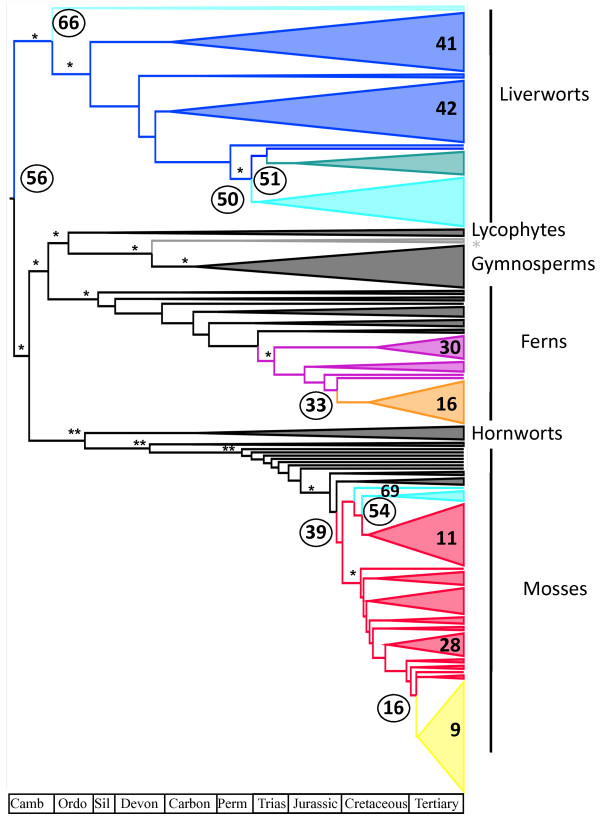
**MEDUSA chronogram**. Diversification chronogram with rate shifts located using MEDUSA [24] for different groups of land plants. Numbers correspond to the rate shifts located by MEDUSA being the numbers in increasing order from the highest to lowest net diversification rate (see Additional file 5). Different colours indicate different net diversification rates found in the tree. Boostrap support for the main nodes are indicated with one asterisk (> 70%) and two asterisk (50%-70%). Angiosperms (grey asterisk) have been simplified for this figure.

Finally, diversification test incorporating multiple birth and death models as implemented in MEDUSA [24] located 69 diversification rate shifts being the highest overall net diversification rates for different clades within angiosperms (Figure 3, Additional file 5). Among land plants we also found rate shifts leading to high clade-size in mosses and ferns for individual families and clades (Figure 3, Additional file 5). On other hand rates among liverworts and gymnosperms were among the lowest: their background rate were similar to the overall background rate, and their highest rates were lower than most rates found for mosses and ferns (see Additional file 5). Results using the constrained tree were widely congruent (Additional file 5) and a new rate shift for gymnosperms and higher net diversification rates across monocots were recovered.

## Discussion and Conclusion

By combining data for all families of land plants we are now able to clarify the picture of their evolution through geological times. Lineages of extant gymnosperms radiated in the Permian and experienced a decrease in diversification rate towards the end of the Jurassic (analysis with unconstrained tree) or early Cretaceous (analysis with constrained tree), during a cool climate mode. Although their early history may have involved various lineage replacements associated with the evolution of new ecosystems [25,26], we found that the slowdown in diversification of gymnosperms took place in the same period as liverworts while mosses were diversifying intensely, pointing towards a role of climate in determining such patterns.

In this study we were also able to evaluate the diversification dynamics of all families of mosses within a phylogenetic framework for the first time. Our analyses converge to show that the diversification rate of this group experienced an important acceleration in the Cretaceous, potentially 'replacing' the diversity of gymnosperms and liverworts. This occurred during a warm climate mode when tropical habitats were undergoing expansion. Significantly, it also corresponds to the origin of the angiosperms according to Brenner [18], or to the origin of major groups of angiosperms (asterids, rosids) as found on our unconstrained analysis and as suggested by previous studies [27]. In this sense, mosses have diversified at the same period as the one reported for fern as "the shadow of angiosperms". It is important to note that the Cretaceous could be divided in three main intervals with regards to vegetation and climate: i) "Early" Cretaceous (ca. Berriasian--Barremian) with few angiosperms, probably no closed canopy angiosperm forests, largely dry climates at low palaeolatitudes; ii) mid-Cretaceous (ca. Aptian-Santonian/Campanian), where we observe the rapid diversification of angiosperms, with presence of some angiosperm-dominated forests but still no tropical everwet forests at low palaeolatitudes; and iii) "Late" Cretaceous (ca. Campanian-Maastrichtian), where we see an early development of angiosperm dominated forests, possibly with everwet forests in low palaeolatitudes of the Old World, and perhaps also in the New World [14]. This is then followed by iv) the Early Cenozoic, when temperatures were warm and climate wet--and where there is strong evidence of widespread tropical-sub-tropical warm wet forests [14]. We found that all six shifts in diversification for mosses (Table 1) fall within this last two intervals (i.e. 75, 69, 64, 57, 43 and 37 mya; see details in Additional files 3), pointing to the importance of both warm temperatures and wet climate for the rise of moss diversity.

According to our analyses, mosses were not the only group to have diversified in the shadow of angiosperms: ferns have also radiated in a period that coincides with the rise of angiosperms. Such a pattern, had previously been reported [28]. Here, we find further support for this hypothesis of diversification in the shadow of angiosperms, identifying a significant increase in diversification during the warmest period of the Cretaceous, and decrease during the coolest period of the Tertiary (see Figure 1). More specifically, three out of the five rate shifts (Table 1) fall within interval iii) above of the Cretaceous, when climate was warm and wet.

Finally, we found that angiosperm diversity has accumulated sharply in recent time (as shown by the LTT plots), but diversification decreased in the coolest period of the Tertiary (Figure 1). This is in agreement with the idea that angiosperms have outcompeted and outnumber gymnosperms and free-sporing plants [29,30]. Subsequently, ferns (especially polypods [28,31]) and mosses [32] opportunistically diversified in the ecological niches provided by the angiosperms as the climate became warmer and more humid. In this sense, our study favours the "successive replacement" of ancestral lineages [14].

## Methods

### Phylogeny

We put together phylogenetic data for at least one representative of each of the 706 currently accepted families of land plants (Additional file 1). Our dataset was assembled using plastid *rbcL*, *atpB *and *rps4 *genes, as well as *18S *and *26S *nuclear ribosomal regions (hereafter *18S *rDNA and *26S *rDNA). We downloaded sequences from GenBank when available and filled some of the gaps by sequencing missing taxa when we were able to obtain suitable material (Additional file 1). DNA extraction and PCR amplification used standard protocols and primers for nuclear and plastid genes from Nickrent and Starr [33] and Cox et al. [34]. We sequenced the *18S *rDNA for 22 angiosperms and 13 mosses, *rbcL *for two angiosperms, 10 mosses and one liverwort, and *atpB *for 39 mosses, two liverworts, one hornwort, and 18 angiosperms (Additional file 1). In total we produced a 6,950 base pairs data matrix consisting of 699 families (including four outgroups) with 65% of data presence. Only one gene could be obtained for 55 of these 699 families (Additional file 1). Streptophytes and Chlorophytes were used as the outgroup.

Due to the large size of the matrix, maximum likelihood analyses were performed in RAxML [35] using 200 bootstrap replicates and GTR+GAMMA model, as selected by ModelTest [36]. Divergence times were calculated using penalized likelihood in r8s [37] and the smoothing parameter (smooth = 1000) was calculated by cross-validation. We calibrated the chronogram with the age of eudicots at 121 mya, corresponding to the appearance of the tricolpate pollen grain typical of this clade [38]. We used a further sixteen calibration points as minimum constrains, plus a maximum age of 725 Ma [39] for the root of the tree (Marchantiopsida, Monilophytes, Mosses, Seed plants, Annonaceae, Calycanthaceae, Hedyosmum, Lauraceae, Magnoliaceae, Meliosma, Menispermaceae, Nelumbaceae, Nymphaceae, Platanaceae, Trochodendron, Winteraceae, Additional file 6). Confidence intervals (C.I.) for divergences times were calculated by repeating the dating procedures in r8s using 100 bootstrapped matrices produced in RAxML [35]. The dating procedure was repeated constraining the age of angiosperms to a maximum of 130 Ma following Brenner [18], i.e. "constrained tree".

### Diversification tests

We examined diversification through time using several methods.

Firstly, we plotted the number of lineages through time (hereafter LTT plots) for each major subclade of land plants using the APE 1.8 package [40].

Secondly, to take into account extinction rates we used the approach of Magallón and Sanderson [19]. For time intervals of one million year, we calculated net diversification rates under a relative high level of background extinction (0.9 using equation 10 of Magallón and Sanderson [19].

Thirdly, we applied a topological-based test of diversification. Diversification rate shifts were calculated using the Δ1 statistics of Moore et al. [20] as implemented by Bouchenak-Khelladi et al. [41] in ApTreeshape [42] using 0.05 significance level as the cut-off point. This test uses the tree but also takes into account the total number of species per family (Table 1).

Fourthly, we used a test of diversification that takes into account branch lengths, i.e. the elapsed time between the nodes of the family-level tree, LASER [23]. Using the Akaike Information Criterion (AIC), LASER can compare models with various rates of diversifications (yule model with rates r) against the null expectation of a constant rate (birth-death model with no extinction). LASER also allows to identify at which points in time a given rate shift occurred (ts). LASER was applied to all major subclades.

Fifthly, we tested for multiple shifts in birth and death rates using a stepwise approach implemented in MEDUSA until improvement in AIC score was < 4 [24]. Net diversification rates together with relative extinction rates and AIC improvements were retrieved (Additional file 5).

Also, to comply with other phylogenetic analyses that have combined more genes but for fewer taxa, we also re-ran the analyses above with the following two modifications. First 11 families for which we could not obtain any DNA data (i.e., five families of liverworts, three of mosses, and three of angiosperms; Additional file 1) were placed in the DNA-based phylogenetic tree using taxonomic information following Crosby et al. [8], Buck and Goffinet [9], Stevens [13], Heinrichs et al. [7] and Smith et al. [11] (see Additional file 2). Although this procedure is suboptimal, it allowed us to perform diversification tests on a complete-family level tree. Second we enforced hornworts and lycophytes to be sister to vascular plants [15,16] plus we set the maximum age for angiosperms to 130 Ma (following Brenner [18]; our "constrained tree"). Results were compared for the constrained vs. unconstrained topologies. Finally, we compared these diversification profiles and metrics against the distribution of the climate modes of the Panerozoic following Frakes et al. [21], as well as the global temperature model of Scotese [22].

## Authors' contributions

OFP carried out the lab work, assembled the data and performed the analyses. OFP, HS and VS designed the study. JH provided crucial material and information. OFP, HS and VS coordinated the study and wrote the manuscript. All authors read and approved the final manuscript.

## Supplementary Material

Additional file 1 **Taxa and GenBank accession numbers with new sequences generated for this study in bold**. Families for which DNA data could not be obtained are indicated in italics. Click here for file

Additional file 2 **Chronogram of the unconstrained tree**. Numbers after family name are species number considered for our analysis following Stevens [13] for angiosperms and gymnosperms, Crosby et al. [8] and Buck and Goffinet [9] for mosses, Smith et al. [11] for ferns and Stotler et al. [43] for liverworts. The x axis indicate time in million years. The placement of 11 families in which no molecular data could be collected are indicated; they were connected to node numbers as follows: 1 - Monocarpaceae, 2 - Sandeothallaceae, 3 - Chonecoleaceae, 4 - Grolleaceae, 5 - Trichotemnomaceae, 6 - Viridivelleraceae, 7 - Microtheliaceae, 8 - Sorapillaceae, 9 - Hapthantaceae, 10 - Balanophoraceae and 11 - Rafflesiaceae. Click here for file

Additional file 3 **Number of significant shift in net diversification rate (topology-based method of Moore et al**. [20]**) with indication of cool (grey) and warm (black) temperatures of the Phanerozoic **[22]. Bold number columns correspond to periods of rate increases. Time unit is Mya (million years ago). For each lineage the bottom row (italics) corresponds to the constrained tree. Click here for file

Additional file 4 **LASER analysis for the constrained tree using a constant-rate birth-death model with no extinction (a = 0) against variable-rates models with 2 and 3 rates (r) and 1 or 2 time shifts given for best fitting model (ts; time unit is million years ago)**. Click here for file

Additional file 5 **Diversification rate shift retrieved from MEDUSA **[24]. Numbers on the first column correspond to the net diversification rate from highest to lowest and are depicted on Figure 3. Non-angiosperms cases are highlighted in bold, followed by the name of the group they belong to in brackets. "r" are the estimates for net diversification rate, "e" are estimates for relative extinction rate, "ΔAIC" is the increase on the stepwise AIC procedure and "ΔAICc" is the increase when corrected for small sample size [24]. Results for the constrained tree are presented at the bottom. Click here for file

Additional file 6 **Minimum-age calibration points used in divergence time reconstructions **[44-57] . Click here for file
