# Supplementary figures and images for: Diversification of land plants: insights from a family-level phylogenetic analysis
Source: BMC Evol Biol. 2011 Nov 21;11:341. doi: 10.1186/1471-2148-11-341 (PMC3227728; doi:10.1186/1471-2148-11-341)

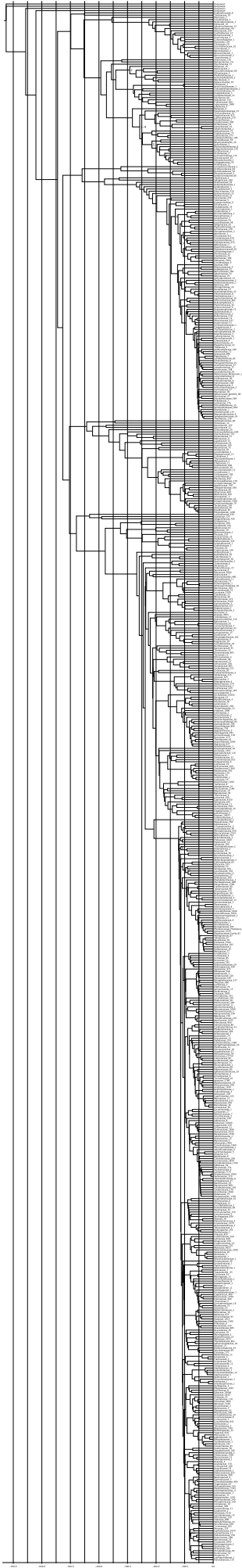

Supplement: Additional file 2 — Chronogram of the unconstrained tree. Numbers after family name are species number considered for our analysis following Stevens [13] for angiosperms and gymnosperms, Crosby et al. [8] and Buck and Goffinet [9] for mosses, Smith et al. [11] for ferns and Stotler et al. [43] for liverworts. The x axis indicate time in million years. The placement of 11 families in which no molecular data could be collected are indicated; they were connected to node numbers as follows: 1 - Monocarpaceae, 2 - Sandeothallaceae, 3 - Chonecoleaceae, 4 - Grolleaceae, 5 - Trichotemnomaceae, 6 - Viridivelleraceae, 7 - Microtheliaceae, 8 - Sorapillaceae, 9 - Hapthantaceae, 10 - Balanophoraceae and 11 - Rafflesiaceae. [file 1471-2148-11-341-S2.PDF]
