# Supplementary material for: Diversification of land plants: insights from a family-level phylogenetic analysis
Source: BMC Evol Biol. 2011 Nov 21;11:341. doi: 10.1186/1471-2148-11-341 (PMC3227728; doi:10.1186/1471-2148-11-341)
Supplement: Additional file 3 — Number of significant shift in net diversification rate (topology-based method of Moore et al. [20]) with indication of cool (grey) and warm (black) temperatures of the Phanerozoic [22]. Bold number columns correspond to periods of rate increases. Time unit is Mya (million years ago). For each lineage the bottom row (italics) corresponds to the constrained tree. [file 1471-2148-11-341-S3.DOC]

**Additional file 3 (Microsoft Word) –** Number of significant shift in net diversification rate (topology-based method of Moore et al. [20]) with indication of cool (black) and warm (bold) temperatures of the Phanerozoic [22].

| Climate modes | 0–34 | **35–133** | 134–156 | **157–257** | 258–309 | **310–427** | 428-444 | Total |
| --- | --- | --- | --- | --- | --- | --- | --- | --- |
| Angiosperms | 5 *5* | **85 *96*** | 2 – | **7 –** | – – | **– –** | – – | 99 *101* |
| Ferns | – – | **5 *4*** | 1 *1* | **1 *1*** | 1 *1* | **1 *2*** | 1 – | 10 *9* |
| Mosses | – *1* | **10 *15*** | 1 *1* | **– –** | – – | **– –** | – – | 11 *17* |
| Gymnosperms | – – | **– *1*** | 1 – | **– –** | – – | **– –** | – – | 1 *1* |
| Liverworts | – – | **– –** | – – | **7 *6*** | 4 *3* | **2 *1*** | 1 *1* | 14 *11* |
| Total | 5 *6* | **100 *116*** | 5 *2* | **15 *7*** | 5 *4* | **3 *3*** | 2 *1* | 135 *139* |

Bold number columns correspond to periods of rate increases. Time unit is Mya (million years ago). For each lineage the bottom row (italics) corresponds to the constrained tree.
