# Supplementary material for: Diversification of land plants: insights from a family-level phylogenetic analysis
Source: BMC Evol Biol. 2011 Nov 21;11:341. doi: 10.1186/1471-2148-11-341 (PMC3227728; doi:10.1186/1471-2148-11-341)
Supplement: Additional file 4 — LASER analysis for the constrained tree using a constant-rate birth-death model with no extinction (a = 0) against variable-rates models with 2 and 3 rates (r) and 1 or 2 time shifts given for best fitting model (ts; time unit is million years ago). [file 1471-2148-11-341-S4.DOC]

**Additional file 4 (Microsoft Word) –** LASER analysis for the constrained tree using a constant-rate birth-death model with no extinction (a=0) against variable-rates models with 2 and 3 rates (r) and 1 or 2 time shifts given for best fitting model (ts; time unit is million years ago).

|  | Birth-death model (a=0) | 2-rates model (r1, r2, ts) | 3-rates model (r1, r2, r3, ts1, ts2) |
| --- | --- | --- | --- |
| Angiosperms |  |  |  |
| AIC | 23.67926 | 17.98178 | 20.2602 |
| Delta AIC | 0 | 5.69748 | 3.41906 |
| Ts |  | 33.59 |  |
| Ferns |  |  |  |
| AIC | 264.4961 | 246.4983 | 243.5756 |
| Delta AIC | 0 | 17.9978 | 20.9205 |
| Ts1 |  |  | 51.71 |
| Ts2 |  |  | 92.72 |
| Mosses |  |  |  |
| AIC | 364.8736 | 287.971 | 279.2117 |
| Delta AIC | 0 | 76.9026 | 85.6619 |
| Ts1 |  |  | 35.19 |
| Ts2 |  |  | 132.65 |
| Gymnosperms |  |  |  |
| AIC | 108.3016 | 103.0154 | 104.1378 |
| Delta AIC | 0 | 5.2862 | 4.1638 |
| Ts |  | 125.33 |  |
| Liverworts |  |  |  |
| AIC | 408.3115 | 353.9121 | 354.4082 |
| Delta AIC | 0 | 54.3994 | 53.9033 |
| Ts |  | 99.52 |  |
