# Supplementary material for: Diversification of land plants: insights from a family-level phylogenetic analysis
Source: BMC Evol Biol. 2011 Nov 21;11:341. doi: 10.1186/1471-2148-11-341 (PMC3227728; doi:10.1186/1471-2148-11-341)
Supplement: Additional file 6 — Minimum-age calibration points used in divergence time reconstructions [44-57] . [file 1471-2148-11-341-S6.DOC]

**Additional file 6 (Microsoft Word) –** Minimum-age calibration points used in divergence time reconstructions [44-57].

| Taxon | Minimum constrain | Reference |
| --- | --- | --- |
| Annonaceae | 69.5 | Chesters 1955 |
| Calycanthaceae | 104 | Friis et al. 1994a |
| Hedyosmum | 118 | Friis et al.1994b |
| Lauraceae | 104 | Crane et al. 1994 |
| Magnoliaceae | 99 | Dilcher and Crane 1984 |
| Meliosma | 69.5 | Magallon et al. 1999 |
| Menispermaceae | 69.5 | Magallon et al. 1999 |
| Nelumbaceae | 100 | Magallon et al. 1999 |
| Nymphaceae | 118 | Friis et al. 2001 |
| Platanaceae | 108 | Magallon et al. 1999 |
| Trochodendron | 14.5 | Manchester et al. 1991 |
| Winteraceae | 121 | Doyle et al. 1990a,b |
| Marchantiopsida | 225 | Heinrichs et al. 2007 |
| Monilophytes | 354 | Bateman 1991 |
| Mosses | 380.44 | Newton et al. 2006 |
| Seed plants | 310 | Miller 1999 |
